# Bengamides display potent activity against drug-resistant *Mycobacterium tuberculosis*

**DOI:** 10.1038/s41598-019-50748-2

**Published:** 2019-10-07

**Authors:** Diana H. Quan, Gayathri Nagalingam, Ian Luck, Nicholas Proschogo, Vijaykumar Pillalamarri, Anthony Addlagatta, Elena Martinez, Vitali Sintchenko, Peter J. Rutledge, James A. Triccas

**Affiliations:** 1 Discipline of Infectious Diseases and Immunology, Faculty of Medicine and Health, The University of Sydney, Camperdown, NSW Australia; 2 Tuberculosis Research Program, Centenary Institute, Sydney, NSW Australia; 3 Charles Perkins Centre, The University of Sydney, Camperdown, NSW Australia; 4 School of Chemistry, Faculty of Science, The University of Sydney, Camperdown, NSW Australia; 5 Centre for Chemical Biology, Indian Institute of Chemical Technology, Secunderabad, India; 6 Centre for Infectious Diseases and Microbiology, The Westmead Institute, Westmead, NSW Australia; 7 Marie Bashir Institute for Infectious Diseases and Biosecurity, The University of Sydney, Camperdown, NSW Australia

**Keywords:** Drug development, Antibiotics

## Abstract

*Mycobacterium tuberculosis* infects over 10 million people annually and kills more people each year than any other human pathogen. The current tuberculosis (TB) vaccine is only partially effective in preventing infection, while current TB treatment is problematic in terms of length, complexity and patient compliance. There is an urgent need for new drugs to combat the burden of TB disease and the natural environment has re-emerged as a rich source of bioactive molecules for development of lead compounds. In this study, one species of marine sponge from the *Tedania* genus was found to yield samples with exceptionally potent activity against *M. tuberculosis*. Bioassay-guided fractionation identified bengamide B as the active component, which displayed activity in the nanomolar range against both drug-sensitive and drug-resistant *M. tuberculosis*. The active compound inhibited *in vitro* activity of *M. tuberculosis* MetAP1c protein, suggesting the potent inhibitory action may be due to interference with methionine aminopeptidase activity. *Tedania*-derived bengamide B was non-toxic against human cell lines, synergised with rifampicin for *in vitro* inhibition of bacterial growth and reduced intracellular replication of *M. tuberculosis*. Thus, bengamides isolated from *Tedania sp*. show significant potential as a new class of compounds for the treatment of drug-resistant *M. tuberculosis*.

## Introduction

The *M. tuberculosis* bacillus is extremely hardy and intrinsically resistant to acid, alkali, lysozymes, osmotic lysis and antibiotic killing^1–3^. This high tolerance to stress is due to the impervious quality of the mycobacterial cell wall, which is hydrophobic and contains few porins, thus reducing rates of transport of hydrophilic molecules and antibiotics into the bacillus^4,5^. For these reasons, effective antibiotics for tuberculosis (TB) are difficult to develop. The current treatment for TB consists of combinations of rifampicin (RIF), isoniazid (INH), ethambutol (EMB) and pyrazinamide (PZA), taken from twice-weekly to daily, over a period of six to nine months^6^. The persistence of infection causes many issues with patient adherence to the regimen and poses a significant obstacle to successful treatment, particularly in areas where the infrastructure required to ensure reliable drug supply, correct prescription and dedicated patient follow-up may be lacking. Intermittent and incomplete treatment increases the risk of relapse and incidence of multiple (MDR-TB) and extensively drug-resistant TB (XDR-TB)^7^, so there is an urgent and pressing need to develop new drugs which can shorten and simplify TB treatment in order to combat the burgeoning MDR-TB pandemic.

Since the golden era of antibiotic discovery during the 1940s and 1950s, when more than 20 new classes of antibiotics entered clinical use, only two novel classes have been discovered^8^. Many current antibiotics are at their sixth or seventh generation of analogue development, and even these applications and approvals have declined steadily over time. One major factor behind the dearth of new antibiotics is the large but generally unsuccessful investment in genomic and target-based approaches by the pharmaceutical industry^9^. The failure of these approaches is likely due to difficulties in translating activity against a cell-free target to potency in more clinically relevant terms (such as the inhibition of whole cells), linked to adverse solubility or metabolic stability. Furthermore, the process of drug discovery and development is lengthy and expensive: it can take more than ten years and cost between US$800M and US$1Bn per drug^10^. The first-line treatment regimen for TB has not been updated to include new drugs for more than 50 years^11^.

Throughout history, the vast majority of antibiotics have been sourced from nature. The first antibiotic used in a clinical setting was pyocyanase, derived from *Pseudomonas aeruginosa*^12^. Due to the extraordinary diversity and hit rates found when screening natural products^13^, there is currently increasing interest in returning to natural sources for drug discovery^14^. The marine environment has come under scrutiny as a relatively untapped cornucopia of novel chemical scaffolds displaying potent bioactivity^15^. An estimated 25% of all Earth’s biodiversity lies in marine species^16^ and an estimated 3.7 × 10^30^ microorganisms live in the marine environment^17^. For example, marine actinomycetes are unique to the ocean environment and produce numerous chemical metabolites distinct from those of terrestrial species^18^. Invertebrate species such as sea sponges and tunicates are other sources of novel drugs, including the FDA-approved cancer drugs trabectedin^19^, cytarabine^20^ and eribulin^21^ and the antiviral vidarabine^22^. In the search for new TB drugs, the marine environment offers a promising avenue of inquiry. In this study, by mining marine samples we have identified bengamide B as a TB drug development lead, being highly potent, synergistic with existing TB drugs and capable of inhibiting growth of intracellular and drug-resistant *M. tuberculosis*.

## Results

### Identification of *M. tuberculosis* inhibitors by screening of marine samples

**Figure 1:**
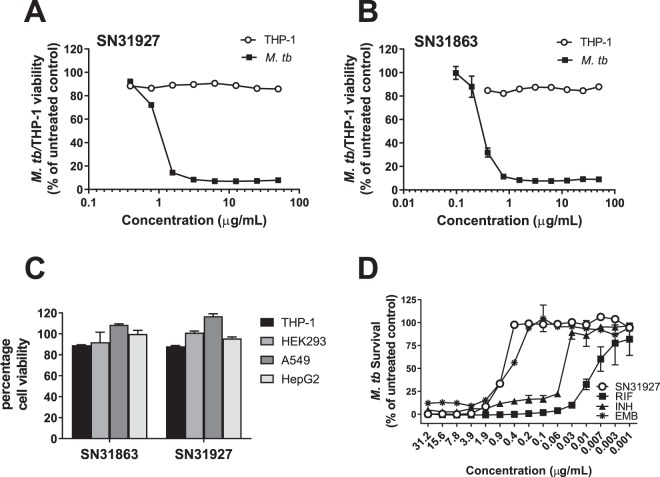
Screening of marine samples to identify potent, non-cytotoxic inhibitors of *M. tuberculosis* H37Rv. Lead samples SN31927 **(A)** and SN31863 **(B)** were incubated with *M. tuberculosis* H37Rv (OD_600nm_ 0.001) or THP-1 cells (2 × 10^5^ cells/well) and after a 5-day incubation resazurin (0.05%) was added and fluorescence measured. Graphs represent percentage viability of bacteria or cells compared with nontreated cells. The viability of HEK293, A549 and HepG2 cell lines was also assessed after incubation with 50 μg/ml crude extract and use of resazurin (0.05%) to calculate cellular viability **(C**). *M. tuberculosis* H37Rv was incubated with varying concentrations of SN31927 extract or two front-line TB^,^ drugs, rifampicin (RIF) or isoniazid (INH), and bacterial viability determined after 5 days incubation **(D**). For all panels data show mean viability ± SEM of triplicate wells and is representative of two independent experiments.

As a first step in the development of TB drug leads, marine samples with inhibitory activity against virulent *M. tuberculosis* H37Rv were identified. To determine this, 1434 diverse marine extracts^23^ were screened for their ability to inhibit *M. tuberculosis* growth *in vitro*. This identified 18 antimycobacterial hits, 11 of which derived from the Porifera phylum and five from the Chordata phylum (Table 1). MIC_50_ determination, defined as the concentration which resulted in 50% survival of bacteria in comparison to untreated controls, revealed that activity range was as low as 0.39 µg/mL (SN31863) and 1.56 µg/mL (SN31927). Samples were screened for toxicity against differentiated THP-1 macrophages by determining CC_50_ values (the concentration at which cellular viability was reduced by 50%) (Table 1). While some samples displayed general toxicity, with comparable MIC_50_ and CC_50_ values (e.g. SN3222), the two most effective antibacterial samples, SN31863 and SN31927, were non-toxic to THP-1 cells over a range of concentrations tested (Fig. 1A,B). As shown in Fig. 1C, extracts SN31863 and SN31927 were also non-cytotoxic against other cell lines tested, including hepatocyte cells (HepG2), epithelial lung cells (A549) and kidney cells (HEK293), suggesting that these compounds have some specificity for mycobacteria. The activity of SN31927 was similar to ethambutol (EMB) when testing in parallel with front-line TB drugs (Fig. 1D).

**Table 1:** Inhibitory activity of lead marine extracts against *M. tuberculosis* and mammalian cell lines.

Source^a^	Sample Number	Type	*M.tb*^b^ Viability (%)	MIC_50_ (µg/mL)	THP-1 Viability (%)	CC_50_ (µg/mL)^c^
Pterobranchia sea squirt	SN32222	Chordata extract	0.25	12.5	9.45	6.25
	SN32228	Chordata 75MeOH eluent	0.25	25	9.2	12.5
	SN32219	Chordata 100MeOH eluent	0.31	50	47.0	50
Halichondriidae sea sponge	SN30916	Porifera 30MeOH eluent	0.37	50	111.61	—
Demospongiae sea sponge	SN30962	Porifera 100MeOH eluent	0.75	50	104.21	—
Ascidian sea squirt	SN30624	Chordata extract	1.09	50	30.5	50
*Tedania* sea sponge	SN31863	Porifera extract	2.85	0.39	87.8	—
	SN31927	Porifera 100MeOH eluent	1.17	1.56	85.7	—
Chalinidae sea sponge	SN31058	Porifera 100MeOH eluent	4.25	50	93.8	—
Demospongiae sea sponge	SN31025	Porifera 30MeOH eluent	7.11	50	92.6	—
Thorectidae sea sponge	SN40000	Porifera extract	14.38	25	8.0	50
Ascidian sea squirt	SN30672	Chordata 75MeOH eluent	23.01	50	50.5	50
*Desmacidon* sea sponge	SN32162	Porifera extract	33.62	12.5	67.8	50
Dictyoceratida sea sponge	SN30623	Chordata extract	35.03	50	50.2	50
*Agelas mauritiana* sea sponge	SN32374	Porifera extract	38.2	50	107.5	—
*Ircinia* sea sponge	SN32265	Porifera 100MeOH eluent	38.24	50	6.5	50
Dictyoceratida sea sponge	SN40074	Porifera MeOH:DCM eluent	45.95	50	97.5	—
*Cymbastela* sea sponge	SN65457	Porifera 50MeOH eluent	46.99	50	102.28	—

^a^Source organisms have not all been identified to species level. Available taxonomic information and common names are shown.

^b^Percentage viability was calculated in comparison to the average of untreated control wells after normalising for background readings.

^c^CC_50_:concentration at which cellular viability was reduced by 50%.

Both SN31927 and SN31863 were derived from a *Tedania* sp. sea sponge collected from the East Diamond Islet of the Tregrosse Reefs, a Coral Sea reef off the coast of Queensland, Australia. Previously reported antibacterial activity of *Tedania* species has been associated with high concentrations of sequestered metals, particularly cadmium and zinc^24^. However, inductively coupled plasma mass spectrometry (ICP-MS) revealed that insignificant amounts of cadmium (40 ppm) or zinc (0.037 ppm) were present in SN31863, the crude sponge extract, compared to values of 2000–15,000 ppm cadmium and 5000–5100 ppm zinc reported by Capon *et al*.^24^. This suggests that the antimycobacterial activity exhibited by samples SN31927 and SN31863 is due to a bioactive compound produced by the organism, and not general metal toxicity.

### Purification of lead samples and characterisation of active fractions

**Figure 2:**
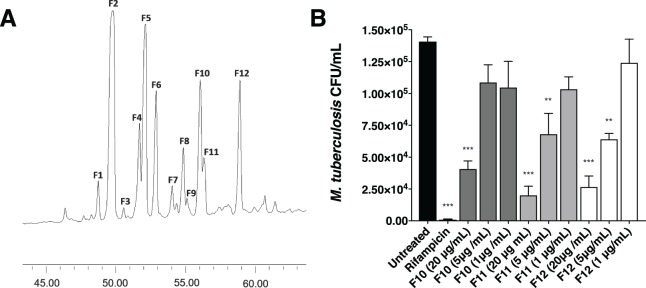
Purification and evaluation of potent, non-cytotoxic inhibitors of *M. tuberculosis* H37Rv. (**A)** Semi-preparative HPLC was carried out on a reversed-phase column (Waters X-bridge C18, 250 mm × 4.6 mm, 5 µm), gradient elution of 0 to 100% acetonitrile–H_2_O, flow rate 1 mL/min over 80 minutes, monitored at 215 nm, with automated fraction collection to yield 12 fractions in the region of interest. **(B)** Intracellular activity assays were conducted by infecting 2 × 10^5^ THP-1 cells/well with 10^6^ *M. tuberculosis* H37Rv for four hours before treatment with F10, F11 or F12 for seven days, lysis of cells and plating for CFU quantifications. Samples were tested in triplicate in two independent experiments. The significance of differences between the untreated groups and other groups were analysed by ANOVA with Tukey’s Multiple Comparisons test **p < 0.01, ****p < 0.0001.

High performance liquid chromatography (HPLC) of SN31863 yielded 12 fractions, F1 to F12 (Fig. 2A), nine of which were antimycobacterial and non-cytotoxic (Table 2). F2 did not inhibit *M. tuberculosis* H37Rv, while insufficient material was recovered from F7 and F9 for detailed bioassay analysis. MIC_50_ determination identified F10, F11 and F12 as the most potent, with F11 and F12 both active down to the low nanogram range (0.078 µg/ml) (Table 2). Encouragingly, all 3 samples displayed no toxicity for the four mammalian cell lines tested (Table 2). To further characterise these 3 fractions, their intracellular antimycobacterial activity was assessed using *M. tuberculosis* H37Rv-infected THP-1 macrophages. All 3 samples significantly inhibited intracellular bacterial growth in a dose-dependent manner (Fig. 2B). Testing F10, F11 and F12 against clinical isolates of drug-resistant *M. tuberculosis* showed that all 3 maintain their potent activity against INH-resistant and RIF/INH-resistant *M. tuberculosis* (Table 3). These findings indicate that the mechanism of action of the *Tedania*-derived compound(s) is likely different to those of existing TB drugs, and as such, these fractions are promising drug leads.

**Table 2:** Antimycobacterial activity and cytotoxicity of purified *Tedania* sp. fractions.

Fraction	*M. tb* viability^a^ (%)	*M. tb* MIC_50_ (μg/mL)	THP-1 viability (%)	HepG2 viability (%)	HEK293 viability (%)	A549 viability (%)
F1	11.4	0.625	88.5	98.9	94.7	101.4
F3	26.7	0.625	94.5	96.9	90.8	107.4
F4	31.4	2.5	94.5	98.8	94.4	105.9
F5	32.9	2.5	51.5	101.4	95.6	63.1
F6	30.9	2.5	90.5	99.3	92.9	105.4
F8	19.3	1.25	91.0	98.0	104.2	103.9
F10	7.8	0.156	92.9	111.7	105.3	105.9
F11	3.0	0.078	91.1	112.2	99.7	101.2
F12	1.3	0.078	90.5	110.8	106.3	100.6

^a^Percentage viability was calculated in comparison to the average of untreated control wells after normalising for background readings.

**Table 3:** Inhibition induced by purified *Tedania* sp. fractions against drug-resistant clinical isolates of *M. tuberculosis*.

Fraction	*M. tb* MIC (µg/mL)	INH-resistant *M.tb* MIC (µg/mL)	RIF/INH-resistant *M. tb* MIC (µg/mL)	RIF/INH/EMB-Resistant MIC (µg/mL)
F10	0.24	0.08	0.08	0.24
F11	0.08	0.08	0.03	0.74
F12	0.01	0.01	0.01	0.08
RIF	0.01	0.04	>2.56	>2.56
INH	0.06	>1.28	>1.28	0.32
EMB	1.28	0.64	0.64	2.56

INH, isoniazid; RIF, rifampicin; EMB, ethambutol.

### Structure elucidation and identification of bioactive molecules

**Figure 3:**
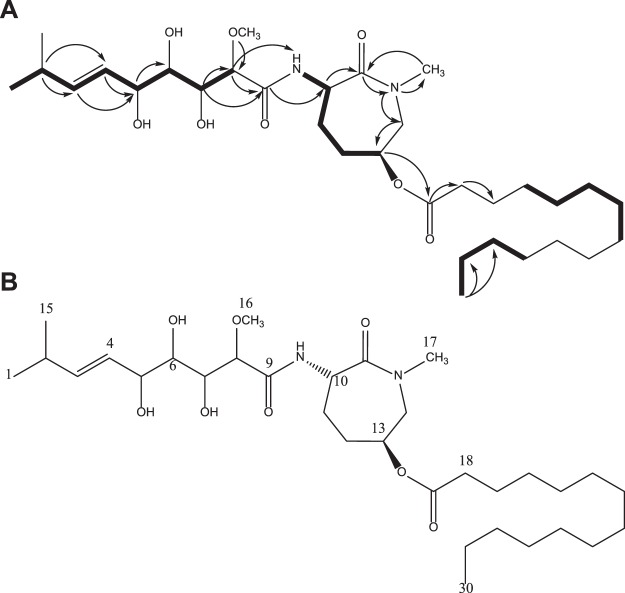
Structure elucidation of F12. (**A**) COSY correlations measured in the current study are shown using bold lines. HMBC correlations are shown using arrows. Some HMBC and COSY correlations have been omitted for clarity. **(B)** Chemical structure of F12 and bengamide B derived from *Jaspis* cf. *coriacea*.

Structure elucidation efforts focused on F12, the most cleanly eluted fraction. Analysis by HRMS-ESI indicated a molecular formula of C_32_H_58_N_2_NaO_8_ [M + Na]^+^ (0.1 ppm) from the adduct ion, and five DBRE were calculated from this formula (Fig. S1). F12 was then characterised by NMR (Figs S2 and S3). ^13^C NMR was used to identify the four multiple bonds (Table S1) as an ester (δ 172.65), two amides (δ 172.31, 170.33) and an (*E*)-CHCH = CHCH array (δ 138.56, 128.04). The presence of a single ring was deduced to be the fifth DBRE apparent in the molecular formula. Additional functional groups included three CH(OH) groups (δ 73.22, 72.94, 71.18), a CH(O-CH_3_) group (δ 81.95, 57.77), a CH flanked by a carbonyl group and an amide NH (δ 51.16, 172.31), a second amide NH connected to a CH_3_ (δ 52.7, 36.11), two CH_2_ groups (δ 138.56, 128.04) and an aliphatic linear chain attached to a carbonyl (δ 172.65, 31.74, 29.49–28.82, 22.75, 14.38). The COSY spectrum contained correlations for C-1 (δ 0.94(7), d) coupled to C-2 (δ 2.15, qqd), which was in turn coupled to C-3 (δ 5.59, ddd), C-4 (δ 5.37, ddd) and C-5 (δ 3.97, dd) (Fig. 3A). The COSY spectrum also revealed the connectivities shown for C-1/C-15 to C-8, C-10 to C-13 and C-18 to C-30 (Fig. 3A). The COSY correlation between C-8 and the methoxy protons revealed the location of the O-CH_3_, and assignment of C-8 adjacent to a carbonyl group was based on both COSY data and HMBC. Other key HMBC correlations included connections between C-9 to H-7 and H-10, C-16 to H10 and N-CH_3_, and C-17 to H-18 (Fig. S4).

Preliminary analysis of these data and comparison with literature indicated close similarity with the previously reported bengamide class of sponge-derived natural products (Fig. 3B). The mass data were in agreement with those in the literature for the known marine heterocycle, bengamide B^25^. Subsequent consideration of the NMR data confirmed this, through diagnostic peaks at δ 5.78, 5.44, 4.60 and 4.21, each of which corresponds to regions of the amide core structure (Fig. 3A). The position and multiplicity of key signals in the ^1^H NMR spectrum of F12 (500 MHz, CDCl_3_) closely matched previously reported data, confirming F12 as bengamide B (Fig. 3B and Table S2).

### Inhibitory activity of bengamide B

Bengamide B from various species inhibits activity of methionine aminopeptidase (MetAP), an enzyme that removes the N-terminal methionine from nascent proteins^26–28^. To confirm this activity in *Tedania*-derived bengamide B, MetAP inhibition against purified enzymes from *M. tuberculosis*, *E. faecalis* and *H. sapiens* was assessed. *Tedania*-derived bengamide B was a highly potent MetAP inhibitor across the 3 different enzymes tested, *Mt*MetAP1c, *Ef*MetAP1b and *Hs*MetAP1b (72.84%, 83.33% and 84.72% inhibition respectively). Interestingly, bengamide B showed strong inhibition of *Ef*MetAP1b, but this was not reflected in the results of bacterial inhibition screening, where bengamide B was not able to inhibit *E. faecalis* growth *in vitro* (data not shown). Similarly, although bengamide B was shown to inhibit *Hs*MetAP1b, it was not cytotoxic against any tested human cell line *in vitro* (Table 2).

### Combination therapy against TB using bengamide B

**Figure 4:**
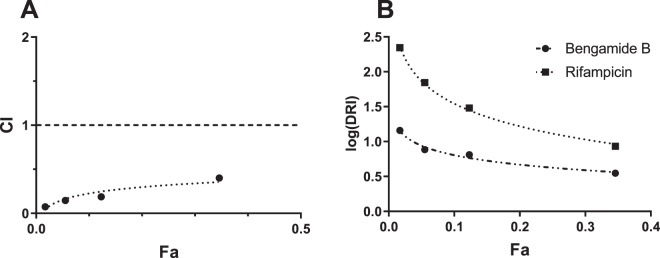
Evaluation of bengamide B and rifampicin synergy. Serial three-fold dilutions of bengamide B (starting conc. 1 μM) were tested in combination with 1 nM rifampicin over four days before the addition of 0.05% w/v/ resazurin overnight. **(A)** Chou-Talalay plot showing CI against Fa. **(B)** Chou-Martin plot showing log(DRI) against Fa.

Multidrug combinations are required to treat TB, and novel drug leads must be able to work in synergy with existing front-line TB drugs. To investigate potential synergistic effects between bengamide B and existing TB drugs, suboptimal concentrations of rifampicin (RIF) (1 nM) were incubated with *M. tuberculosis* H37Rv in combination with bengamide B at a range of concentrations (0.03 μM, 0.11 μM, 0.33 μM, 1 μM). The results were analysed using the Chou-Talalay combination index (CI) equation^29^ and revealed strong synergy between bengamide B and rifampicin (CI = 0.1–0.3) (Fig. 4A). Furthermore, combining bengamide B and rifampicin allows for a dose reduction index (DRI) from 8-fold to over 200-fold for rifampicin and 3-fold to over 14-fold for bengamide B over a range of concentration combinations (Fig. 4B).

## Discussion

Despite the heavy burden of TB disease and growing rates of drug resistance worldwide, efforts to develop new drugs for treatment have met with limited success. The two most recently approved drugs for TB are bedaquiline, discovered through investigation and optimisation of the diarylquinolone compound family^30^ and licensed for use by the FDA in 2012^31^, and delamanid, licensed in 2014^32^. Since then, the treatments which have progressed furthest through clinical trials are simply combinations of existing drugs^33^. Further work is urgently needed to bring novel and overlooked molecular scaffolds through development and into the TB drug pipeline. This study aimed to mine the marine biosphere in the search for potential TB drug leads using whole-cell screening techniques. The results reiterate that marine organisms are rich and underutilised sources of bioactive molecules. The most active antimycobacterial compounds identified in this study derived from sea sponges and sea squirts of the Porifera and Chordata phyla. As sessile filter feeders, these organisms rely heavily on secondary metabolites to gain competitive advantage in the struggle for space and resources in their benthic habitats^34^. Nine samples among the 18 antimycobacterial hits were broadly cytotoxic, and likely contain highly potent biological deterrents specifically produced by the organism, like other well-known examples such as cone shell poison, tetrodotoxin from puffer fish and stonefish neurotoxin^35,36^. Toxicity is the predominant cause of failure in marine natural product drug screening, with the cost of attrition increasing as samples progress through preclinical testing^37^. Consequently, any hits which resulted in cytotoxicity against any of the tested human cell lines were discounted from further investigation.

*Tedania* sea sponges are known to cause blistering and peeling upon contact with bare skin^38^ and are therefore recognised as producers of potent bioactive molecules. *T. ignis* is the *Tedania* species that has been best documented in drug discovery studies, giving rise to several novel indoles^39^, an atisanediol^40^, a δ-lactam^41^, diketopiperazines^39^, the tedanolide family of potent antitumour macrolides^42^, and the tedanols, a group of anti-inflammatory diterpenes^43^. *T. anhelans* has given rise to some interesting pyrazole acids^44^, and *T. digitis* has been documented as the source of tedaniaxanthin^45^. All of these novel molecules and scaffolds isolated from *Tedania* spp. are larger (50–600 g/mol) aromatic compounds with some level of cytotoxicity. To date, the only antimycobacterial agents isolated from *Tedania* sponges were derived from a symbiotic strain of *Penicillium chrysogenum* with broad-spectrum inhibitory activity against *S. aureus*, *P. aeruginosa*, *M. tuberculosis* H37Ra, *M. avium*, *M. smegmatis*, *M. fortuitum*, *M. vaccae*, *Aeromonas hydrophila*, and *Vibrio cholerae*^46^. Purification of the active *Tedania* sp. sponge extract in the current study resulted in 11 active antimycobacterial fractions, one of which was resolved through mass spectrometry and NMR analysis as bengamide B. The other fractions either resulted in too little dry weight of material, or were not sufficiently pure for structure elucidation.

The bengamides are a series of small molecules consisting of a core amide ring, a polyketide chain and an alkyl ester. Originally discovered as antiparasitic compounds in a study of Indo-Pacific sponge extracts, bengamides A and B were first isolated from *Jaspis* cf. *coriacea* sponges in 1986^25^. Since then, the bengamide class of molecules has been found to have nanomolar antitumour activity^47^ due to the inhibition of methionine aminopeptidase (MetAP) type 1 and type 2^28^, arresting cell growth at phases G1 and G2/M^48^. Bengamides have also been found to inhibit the NF-κB pathway, acting as potent anti-inflammatory compounds^49^. However the best-characterised bengamide, bengamide B, has gained most recognition as an antitumour agent. When used to treat MDA-MB435 human breast cancer xenografts on athymic rats *in vivo*, bengamide B significantly inhibits growth of MDA-MB435 human breast cancer xenografts^49^ and analogues of treatment have progressed to clinical trials for treatment of refractory solid phase tumours^50^. Interestingly, despite the levels of tumour cell line inhibition reported for bengamide B in the literature, the bengamide B natural product isolated in the current study did not display any notable cytotoxicity against THP-1 derived macrophages, HepG2, HEK293 or A549 cells (Fig. 1). Further cytotoxicity studies, both *in vitro* and *in vivo*, are required to probe the effects of bengamide B in mammalian and human systems in order to inform the TB drug lead development process. One previous study has attempted to develop TB drug leads by synthesising bengamide derivatives to bind to the four different metalloforms of *Mt*MetAP1a and *Mt*MetAP1c (Co, Mn, Ni, Fe)^27^. The two best-performing derivatives were potent MetAP inhibitors but resulted in modest MIC values of 122 and 53.9 µM respectively against mycobacteria, in contrast to the nM activity observed for *Tedania*-dervied bengamide B (Table 2).

It has been well established that potent target inhibition does not necessarily translate to activity in clinically relevant terms, and target-based approaches to drug design have in fact been blamed for high rates of attrition and low productivity in pharmaceutical research^51^. We observed that while bengamide B is strongly inhibitory against *Mt*MetAP1c, *Ef*MetAP1b and *Hs*MetAP1b, it is not able to inhibit the growth of *E. faecalis*, or THP-1 derived macrophages, HepG2, HEK293 or A549 cells (Table 2). MetAPs are essential enzymes responsible for the cotranslational removal of N-terminal methionine from newly synthesised proteins, triggering localisation, activation or degradation^52^. Found across both prokaryotic and eukaryotic cells, MetAPs have been investigated as attractive anticancer, anti-parasitic, and anti-atherosclerotic drug targets over the years^53^. Bengamides are known to inhibit both MetAP1 and MetAP2 in humans, and it has been postulated that this non-selective activity is responsible for clinical cytoxicity as a result of global N-terminal methionine processing inhibition^26^.

There are no records in the literature to date of evolved resistance against bengamide B, even though the inhibitory properties of bengamide B have been explored in *Streptococcus pyrogenes*, *Nippostrongylus braziliensis* and various human cell lines^25^. However, one genetically engineered mechanism of resistance to MetAP inhibition in *M. tuberculosis* has been reported. The overexpression of either *Mt*MetAP1a or *Mt*MetAP1c in knock-in strains of *M. tuberculosis* confers resistance to the MetAP inhibitor 2,3-dichloro-1,4-naphthoquinone^54^. This reinforces the hypothesis that MetAPs are essential, non-redundant enzymes, though other effects have been reported from MetAP inhibition. For example, the MetAP inhibitors ovalicin and fumagillin are able to inhibit angiogenesis^55,56^. As *M. tuberculosis* is notorious for upregulating host angiogenesis to facilitate bacterial spread from the site of infection^57^, targeting MetAPs is an attractive treatment strategy. No TB drug in current use is known to target *Mt*MetAPs and it is likely that drug-resistant strains have not been selected for resistance to MetAP inhibitors. This has been confirmed experimentally in this study by the demonstrated ability of bengamide B to strongly inhibit multidrug resistant *M. tuberculosis* strains. The results of the microdilution drug combination assay indicate that bengamide B is able to work in synergy with rifampicin to inhibit the growth of *M. tuberculosis* H37Rv (Fig. 4), suggesting bengamide B is a promising candidate to investigate as a means of improving current suboptimal treatment of drug-resistant and drug-susceptible TB.

In conclusion, this study has identified a *Tedania* sp. sea sponge as a source of potent, non-cytotoxic antimycobacterial extracts with activity against clinical strains of MDR-TB. With the critical shortage of novel scaffolds in the TB drug pipeline, all avenues must be explored in the search for new drug leads. This study has reiterated the wealth of bioactive molecules which can be sourced from the marine environment, both from sea sponges and their associated microflora. Future directions include the purification of additional bioactive compounds from the identified *Tedania* extract for characterisation and further testing.

## Materials and Methods

### Bacterial strains, media and culture conditions

*M. tuberculosis* H37Rv (ATCC27294) was used for this study, along with the clinical *M. tuberculosis* 3410 (INH-resistant), 1863 (RIF/INH-resistant) and 2441 (RIF/INH/EMB-resistant) isolates. Middlebrook 7H9 media supplemented with albumin-dextrose-catalase (ADC; 10% v/v), glycerol (0.2% v/v) and Tween-80 (0.05% v/v) was used to grow cultures which were incubated at 37 °C in a humidified 5% CO_2_ incubator.

### Cell lines, media and culture conditions

THP-1 (ATCC TIB-202), HepG2 (ATCC HB-8065), HEK293 (ATCC CRL-1573) and A549 (ATCC CCL-185) cells were used in this study. Frozen cell stocks were taken from liquid nitrogen, thawed and grown in cell culture flasks containing 25 mL RPMI for THP-1 cells, or DMEM for HepG2, HEK293 and A549 cells. All cell media was supplemented with 10% v/v foetal calf serum, 100 U/mL penicillin and 0.1% w/v streptomycin. The flasks were incubated in a 5% CO_2_ incubator at 37 °C until they reached ~80% confluency, at which point the cells were trypsinised if required and counted before resuspension at required cell concentrations.

### Marine samples

Test samples consisted of 239 crude extracts obtained from marine organisms provided by the Australian Institute of Marine Science (AIMS). Crude extracts were sequentially fractionated in methanol by AIMS using a pre-equilibrated solid phase extraction Phenomenex Strata C18-E cartridge (55 μm, 70 Å, 500 mg/6 mL) to generate 1434 samples. All samples were supplied in 100% DMSO solution at 5 mg/mL and stored at -80 °C. The marine sponge *Tedania* sp. was collected from the East Diamond Islet of the Tregrosse Reefs off the coast of Queensland, Australia. The sponge was deep-frozen immediately after collection and freeze-dried before extraction with methanol. This crude extract was then stored at −80 °C.

### Screening for mycobacterial inhibition

The resazurin reduction assay is a quantitative, colorimetric means of determining cell viability and was employed throughout this study to determine growth inhibition^58^. Bacterial strains were grown to log phase and diluted to a stock concentration of OD_600_ = 0.001. Test samples (0.5 mg/mL) or positive controls (5 μM rifampicin) were diluted in dH_2_O in 96 well plates and 10 μL of each sample dispensed into separate wells. Ninety μL of bacterial suspension was added to each well and incubated at 37 °C for 4 days, after which 10 μL of 0.05% resazurin was added to each well. After overnight incubation, fluorescence readings were taken at 590 nm after excitation at 544 nm using the FLUOstar Omega Microplate Reader (BMG Labtech, Ortenburg/Germany). Percentage viability was calculated in comparison to the average of untreated control wells after normalising for background readings. Z’-factors were greater than 0.5 for all assays^59^. For the clinical isolates of drug-resistant *M. tuberculosis*, plates were incubated for 7 days before the addition of resazurin for 24 h and results of the resazurin reduction assay were read by eye. To determine MIC_50_ values, serial two-fold dilutions of test samples were made using dH_2_O to give final sample concentrations of 500 µg/mL to 0.01 µg/mL, which were tested as described above, and MIC_50_ was determined according to percentage survival in comparison to untreated controls. To assess drug synergy, *M. tuberculosis* H37Rv (OD_600_ = 0.001) was added to wells containing suboptimal concentrations of rifampicin (1 nM) or isoniazid (0.25 µM) together with with 1:3 serial dilutions of bengamide B starting at MIC = 1 μM. The plates were incubated and treated with resazurin before fluorescence readings, as described above.

### Screening for cytotoxicity

Cells were seeded into 96-well plates at THP-1 cells (2 × 10^5^ cells/well) were treated with 100 ng/mL phorbol 12-myristate 13-acetate (PMA) before seeding in order to stimulate differentiation into macrophages-like cells. All cells were left to adhere in a 5% CO_2_ incubator at 37 °C for 48 h before test samples were added to a final concentration of 50 µg/mL. Plates were incubated for 4 days, then treated with resazurin for 16 h and read for fluorescence as per the previous section. To determine CC_50_ values, serial two-fold dilutions of test samples were made using dH_2_O to give final sample concentrations of 500 µg/mL to 3.9 µg/mL, which were tested as described above.

### Heavy metal content analysis

Prior to analysis, solid sponge sample was dissolved in concentrated nitric acid, then adjusted to 14% v/v concentration of nitric acid using Milli-Q H_2_O before undergoing microwave digestion and passing through a 0.4 µm syringe filter. Cadmium and zinc standards were prepared by diluting stock standard solutions to levels in the linear range for the instrument using the same acidified Milli-Q water used in the preparation of the sponge digestate. Inductively coupled plasma optical emission spectrometry (ICP-OES) analyses were performed on the Perkin-Elmer OPTIMA 7000 DV Inductively Coupled Plasma-Optical Emission Spectrometer equipped with an axial torch, cyclonic spray chamber, and Meinhard^®^ Type C concentric nebuliser. The operating conditions were RF Power 1300 W, plasma flow 15 L/min, auxiliary flow 0.2 L/min, nebuliser flow 0.8 L/min and a viewing distance of 15.0 mm. Each element was initially analysed by three spectral lines and then a single spectral line that exhibited low interference and high analytical signal and background ratios were selected for each element. These spectral lines were 228.804 nm (Cd) and 213.860 (Zn). Each element was quantified by the average of two readings.

### Purification and identification of inhibitory compounds

Semi-preparative HPLC of *Tedania s*p. sample was carried out on a reversed-phase column (Waters X-bridge C18, 250 mm × 4.6 mm, 5 µm), 100 µL sample injection and a gradient elution with 0 to 100% acetonitrile–H_2_O at 1 mL/min over 80 minutes, monitored at 215 nm. Fractions were collected, freeze dried and dissolved in DMSO at 5 mg/mL for testing against *M. tuberculosis* H37Rv and THP-1 derived macrophages. Electrospray Ionisation (ESI) High Resolution Mass Spectrometry (HRMS) was recorded in a positive ion mode on a Fourier transform ion cyclotron resonance mass spectrometer (Apex Qe 7T, Bruker Daltonics, Bremen, Germany) with an Apollo II electrospray ionisation (ESI) ion source. The solvent for HRMS was CH_3_CN. NMR spectra were recorded on a Bruker AVANCE III 500 (^1^H at 500.13 MHz and ^13^C at 125.21 MHz). Spectra were referenced to residual solvent resonances (CHCl_3_ δ 7.26 (^1^H) and δ 77.16 (^13^C); DMSO δ 2.50 (^1^H) and δ 39.52 (^13^C)). All samples were dissolved in approximately 45 μL of DMSO-*d*6 or CDCl_3_ and transferred to 1.7 mm NMR tubes. All data were collected and processed using Bruker Topspin 3.5 software. Chemical shifts (δ) are reported in ppm, coupling constants in Hz, and for signal multiplicities: s = singlet, d = doublet, dd = doublet of doublet, ddd = doublet of doublet of doublet, dddd = doublet of doublet of doublet of doublet, qqd = quartet of quartet of doublet, t = triplet, m = multiplet.

### Intracellular antimycobacterial activity assay

THP-1 cells were grown to ~80% confluency in cell culture flasks and then counted, dispensed into 96-well flat-bottomed wells at 2 × 10^5^ cells/well and left to differentiate as described above. Cells were then infected with 10^6^ *M. tuberculosis* H37Rv in complete RPMI for four hours. The media was removed and the cells gently washed 3 times with 2% FBS in PBS. One hundred μL complete RPMI was then added to each well along with test samples at a final concentration of 50 µg/mL. Rifampicin at 5 µM was used as a positive control for intracellular inhibition. The plates were incubated at 37 °C in 5% CO_2_ for 7 days, following which cells were lysed with cold dH_2_O and bacteria enumerated by plating on Middlebrook 7H11 agar supplemented with oleic-acid-albumin-dextrose catalase (OADC; 10% v/v) and glycerol (0.5% v/v) for 3 weeks at 37 °C.

### Combination analysis

Synergism between bengamide B and rifampicin was determined by calculating the CI (CI = D1/Dx1 + D2/Dx2, where Dx1 and Dx2 indicate the individual dose of bengamide B and rifampicin required to inhibit a given level of viability index, and D1and D2 are the doses of bengamide B and rifampicin necessary to produce the same effect in combination, respectively). CI values of <1, =1, and >1 indicate synergism, additive effect, and antagonism of drugs, respectively. The DRI for both bengamide B and rifampicin were calculated using the multiple drug effect equation (DRI = Dx1/D1), where DRI values quantify how many folds of dose reduction result from drug combination in comparison to single drug treatment. Both CI and DRI were plotted against fraction affected (FA) to generate a Chou-Talalay and Chou-Martin plot providing visual illustration of synergism and dose-reduction.

### MetAP inhibition

*Mt*MetAP1c, *Ef*MetAP1b and *HS*MetAP1b enzymes were expressed in *E. coli* and purified^60–62^. Samples were tested at a single concentration of 50 µM and enzymatic activity was monitored by fluorescence readings following hydrolysis of the fluorogenic substrate, methionyl aminomethylcoumarin, at room temperature. Percentage inhibition was calculated in comparison to negative controls.

### Statistical analysis

Statistical analysis was performed using GraphPad Prism 6 or 7 software (GraphPad Software, La Jolla, CA). Differences between two groups were analysed by Student’s t-test, or between multiple groups by ANOVA with Tukey’s Multiple Comparisons test and were considered significant when the P values were ≤0.05.

## Supplementary information

Supplementary Information

## Data Availability

Data from this study will be made fully available and without restriction upon request.

## References

[1] Jackett PS, Aber VR, Lowrie DB (1978). Virulence and resistance to superoxide, low pH and hydrogen peroxide among strains of *Mycobacterium tuberculosis*. J Gen Microbiol.

[2] Vandal OH, Nathan CF, Ehrt S (2009). Acid resistance in *Mycobacterium tuberculosis*. J Bacteriol.

[3] Gerston KF, Blumberg L, Tshabalala VA, Murray J (2004). Viability of mycobacteria in formalin-fixed lungs. Hum Pathol.

[4] Brennan PJ, Nikaido H (1995). The envelope of mycobacteria. Annu Rev Biochem.

[5] Trias J, Jarlier V, Benz R (1992). Porins in the cell wall of mycobacteria. Science.

[6] Schaberg T (2015). Treatment of tuberculosis. Current standards. Internist (Berl).

[7] Mahmoudi A, Iseman MD (1993). Pitfalls in the care of patients with tuberculosis. Common errors and their association with the acquisition of drug resistance. JAMA.

[8] Ventola CL (2015). The antibiotic resistance crisis: part 1: causes and threats. P T.

[9] Brotz-Oesterhelt H, Sass P (2010). Postgenomic strategies in antibacterial drug discovery. Future Microbiol.

[10] DiMasi JA, Hansen RW, Grabowski HG (2003). The price of innovation: new estimates of drug development costs. J Health Econ.

[11] WHO. Global Tuberculosis Report 2017 (2017).

[12] Emmerich R, Low O (1899). Bakteriolytische Enzyme als Ursache der erworbenen Immunität und die Heilung von Infectionskrankheiten durch dieselben. Z Hyg Infektionskr.

[13] Sukuru SC (2009). Plate-based diversity selection based on empirical HTS data to enhance the number of hits and their chemical diversity. J Biomol Screen.

[14] Rutledge PJ, Challis GL (2015). Discovery of microbial natural products by activation of silent biosynthetic gene clusters. Nat Rev Microbiol.

[15] Mehbub MF, Perkins MV, Zhang W, Franco CMM (2016). New marine natural products from sponges (Porifera) of the order Dictyoceratida (2001 to 2012); a promising source for drug discovery, exploration and future prospects. Biotechnol Adv.

[16] Mora C, Tittensor DP, Adl S, Simpson AGB, Worm B (2011). How Many Species Are There on Earth and in the Ocean?. PLOS Biology.

[17] Kennedy J (2010). Marine metagenomics: new tools for the study and exploitation of marine microbial metabolism. Mar Drugs.

[18] Subramani R, Aalbersberg W (2012). Marine actinomycetes: an ongoing source of novel bioactive metabolites. Microbiol Res.

[19] Le Cesne A, Reichardt P (2015). Optimizing the use of trabectedin for advanced soft tissue sarcoma in daily clinical practice. Future Oncol.

[20] Chhikara BS, Parang K (2010). Development of cytarabine prodrugs and delivery systems for leukemia treatment. Expert Opin Drug Deliv.

[21] Garrone O (2017). Eribulin in advanced breast cancer: safety, efficacy and new perspectives. Future Oncol.

[22] Whitley R (1991). A controlled trial comparing vidarabine with acyclovir in neonatal herpes simplex virus infection. Infectious Diseases Collaborative Antiviral Study Group. N Engl J Med.

[23] Evans-Illidge EA (2013). Phylogeny drives large scale patterns in Australian marine bioactivity and provides a new chemical ecology rationale for future biodiscovery. PLoS One.

[24] Capon RJ (1993). Extraordinary Levels of Cadmium and Zinc in a Marine Sponge, *Tedania-Charcoti* Topsent - Inorganic Chemical Defense Agents. Experientia.

[25] Quinoa E, Adamczeski M, Crews P, Bakus GJ (1986). Bengamides, Heterocyclic Anthelmintics from a *Jaspidae* Marine Sponge. Journal of Organic Chemistry.

[26] Hu X (2007). Regulation of c-Src nonreceptor tyrosine kinase activity by bengamide A through inhibition of methionine aminopeptidases. Chem Biol.

[27] Lu JP (2011). Inhibition of *Mycobacterium tuberculosis* methionine aminopeptidases by bengamide derivatives. ChemMedChem.

[28] Towbin H (2003). Proteomics-based target identification: bengamides as a new class of methionine aminopeptidase inhibitors. J Biol Chem.

[29] Chou TC, Talalay P (1984). Quantitative analysis of dose-effect relationships: the combined effects of multiple drugs or enzyme inhibitors. Adv Enzyme Regul.

[30] Diacon AH (2009). The diarylquinoline TMC207 for multidrug-resistant tuberculosis. N Engl J Med.

[31] Conradie F (2014). Clinical access to Bedaquiline Programme for the treatment of drug-resistant tuberculosis. S Afr Med J.

[32] Ryan NJ, Lo JH (2014). Delamanid: first global approval. Drugs.

[33] Palomino JC, Martin A (2013). Tuberculosis clinical trial update and the current anti-tuberculosis drug portfolio. Curr Med Chem.

[34] Thakur AN (2005). Antiangiogenic, antimicrobial, and cytotoxic potential of sponge-associated bacteria. Mar Biotechnol (NY).

[35] Wilson DM, Puyana M, Fenical W, Pawlik JR (1999). Chemical defense of the Caribbean reef sponge *Axinella corrugata* against predatory fishes. J Chem Ecol.

[36] Wu ZY, Li YT, Xu DJ (2005). Diaqua(2,2’-diamino-4,4’-bi-1,3-thiazole)oxosulfatovanadium(IV) tetrahydrate. Acta Crystallogr C.

[37] Kola I, Landis J (2004). Can the pharmaceutical industry reduce attrition rates?. Nat Rev Drug Discov.

[38] Isbister GK, Hooper JN (2005). Clinical effects of stings by sponges of the genus *Tedania* and a review of sponge stings worldwide. Toxicon.

[39] Dillman RL, Cardellina JH (1991). Aromatic Secondary Metabolites from the Sponge *Tedania-Ignis*. Journal of Natural Products.

[40] Schmitz FJ (1983). Metabolites from the Marine Sponge Tedania-Ignis - a New Atisanediol and Several Known Diketopiperazines. Journal of Organic Chemistry.

[41] Cronan JM, Cardellina JH (1994). A Novel δ-Lactam from the Sponge *Tedania ignis*. Natural Product Letters.

[42] Chevallier C (2006). Tedanolide C: A potent new 18-membered-ring cytotoxic macrolide isolated from the Papua New Guinea marine sponge *Ircinia* sp. Journal of Organic Chemistry.

[43] Costantino V (2009). Tedanol: A potent anti-inflammatory ent-pimarane diterpene from the Caribbean Sponge *Tedania ignis*. Bioorgan Med Chem.

[44] Parameswaran PS, Naik CG, Hegde VR (1997). Secondary metabolites from the sponge *Tedania anhelans*: Isolation and characterization of two novel pyrazole acids and other metabolites. Journal of Natural Products.

[45] Tanaka Y, Katayama T (1979). Biochemical Studies on the Carotenoids in Porifera: The Structure of Tedaniaxanthin. Nippon Suisan Gakkaishi.

[46] Visamsetti A, Ramachandran SS, Kandasamy D (2016). Penicillium chrysogenum DSOA associated with marine sponge (*Tedania anhelans*) exhibit antimycobacterial activity. Microbiol Res.

[47] Kinder FR (2001). Total syntheses of bengamides B and E. J Org Chem.

[48] Phillips PE (2000). Bengamide E arrests cells at the G1/S restriction point and within the G2/M phase of the cell cycle. Proc Annu Meet Am Assoc Cancer Res.

[49] Johnson TA (2012). Myxobacteria versus sponge-derived alkaloids: the bengamide family identified as potent immune modulating agents by scrutiny of LC-MS/ELSD libraries. Bioorg Med Chem.

[50] Dumez H (2007). A phase I and pharmacokinetic study of LAF389 administered to patients with advanced cancer. Anticancer Drugs.

[51] Swinney DC, Anthony J (2011). How were new medicines discovered?. Nat Rev Drug Discov.

[52] Bradshaw RA, Brickey WW, Walker KW (1998). N-terminal processing: the methionine aminopeptidase and N alpha-acetyl transferase families. Trends Biochem Sci.

[53] Vaughan MD, Sampson PB, Honek JF (2002). Methionine in and out of proteins: targets for drug design. Curr Med Chem.

[54] Olaleye O (2010). Methionine Aminopeptidases from Mycobacterium tuberculosis as Novel Antimycobacterial Targets. Chemistry & Biology.

[55] Griffith EC (1997). Methionine aminopeptidase (type 2) is the common target for angiogenesis inhibitors AGM-1470 and ovalicin. Chemistry & Biology.

[56] Sin N (1997). The anti-angiogenic agent fumagillin covalently binds and inhibits the methionine aminopeptidase, MetAP-2. P Natl Acad Sci USA.

[57] Polena H (2016). *Mycobacterium tuberculosis* exploits the formation of new blood vessels for its dissemination. Sci Rep.

[58] Yu M (2016). Nontoxic Metal-Cyclam Complexes, a New Class of Compounds with Potency against Drug-Resistant *Mycobacterium tuberculosis*. J Med Chem.

[59] Zhang JH, Chung TDY, Oldenburg KR (1999). A simple statistical parameter for use in evaluation and validation of high throughput screening assays. Journal of Biomolecular Screening.

[60] Hu XY, Addlagatta A, Matthews BW, Liu JO (2006). Identification of pyridinylpyrimidines as inhibitors of human methionine aminopeptidases. Angew Chem Int Edit.

[61] Kishor C, Gumpena R, Reddi R, Addlagatta A (2012). Structural studies of *Enterococcus faecalis* methionine aminopeptidase and design of microbe specific 2,2 ‘-bipyridine based inhibitors. Medchemcomm.

[62] Reddi R (2014). Selective targeting of the conserved active site cysteine of *Mycobacterium tuberculosis* methionine aminopeptidase with electrophilic reagents. Febs J.

