# Supplementary material for: Bengamides display potent activity against drug-resistant Mycobacterium tuberculosis
Source: Sci Rep. 2019 Oct 7;9:14396. doi: 10.1038/s41598-019-50748-2 (PMC6779907; doi:10.1038/s41598-019-50748-2)
Supplement: Supplementary file 1 — Supplementary Information [file 41598_2019_50748_MOESM1_ESM.pdf]

Supplementary Information

Bengamides display potent activity against non-replicating and drug-resistant *Mycobacterium tuberculosis*

Diana H. Quan, Gayathri Nagalingam, Ian Luck, Nicholas Proschogo, Vijaykumar Pillalamarri, Anthony Addlagatta, Elena Martinez, Vitali Sintchenko, Peter J. Rutledge and James A. Triccas

Table of Contents

Table S1. *^{1}H NMR Spectroscopic Assignments for F12 in comparison to bengamide B derived from Jaspis cf. coriacea. (500 MHz, $CDCl_3$).* 2

Figure S1. *High resolution ESI mass spectrum of F12* 3

Figure S2. *1H NMR spectrum for F12* 4

Table S2. *13C NMR spectrum of F12* 5

Figure S3. *1H and 13C NMR signals* 6

Figure S4. *Stacked 2D NMR 1H-13C HSQC and HMBC spectra of F12* 7

Bengamide B[a] CDCl$_3$		F12 CDCl$_3$	
δ$_H$ (ppm)	δ$_H$, Multiplicity (J in Hz)	δ$_H$ (ppm)	δ$_H$, Multiplicity (J in Hz)
8.11	d, J = 6.2; 1H	8.10	d, J = 6.13; 1H
5.79	ddd, J = 15.5, 6.5, 0.9; 1H	5.78	dd, J = 15.55, 6.52; 1H
5.46	ddd, J = 15.6, 7.3, 1.3; 1H	5.45	ddd, J = 15.62, 7.19, 1.0; 1H
4.65	m; 2H	4.66	m; 2H
4.28	s; 1H		
4.22	t, J = 6.3; 1H	4.22	t, J = 6.15; 1H
3.67	dd, J = 14.6, 10.1; 1H	3.66	m; 1H
3.80	m; 2H	3.82	m; 2H
3.60	s; 1H	3.60	
3.55	s; 3H	3.54	s; 3H
3.22	m; 2H	3.23	d, J = 4.88; 2H
3.07	s; 1H		
3.11	s; 3H	3.10	s; 3H
2.31	t, J = 7.4; 2H	2.31	t, J = 7.44; 2H
2.15	m; 2H	2.16	m; 2H
1.97	m; 1H	1.97	m; 1H
1.63	m; 4H	1.62	m; 4H
1.26	m; 20H	1.26	m; 20H
1.00	dd, J = 6.8, 2.7; 6H	1.00	dd, J = 6.73, 2.5; 6H
0.88	t, J = 6.8; 3H	0.88	t, J = 6.8; 3H

[a]Data taken from[44]

Table S1. ^{1}H NMR Spectroscopic Assignments for F12 in comparison to bengamide B derived from *Jaspis* cf. *coriacea*. (500 MHz, CDCl$_3$).

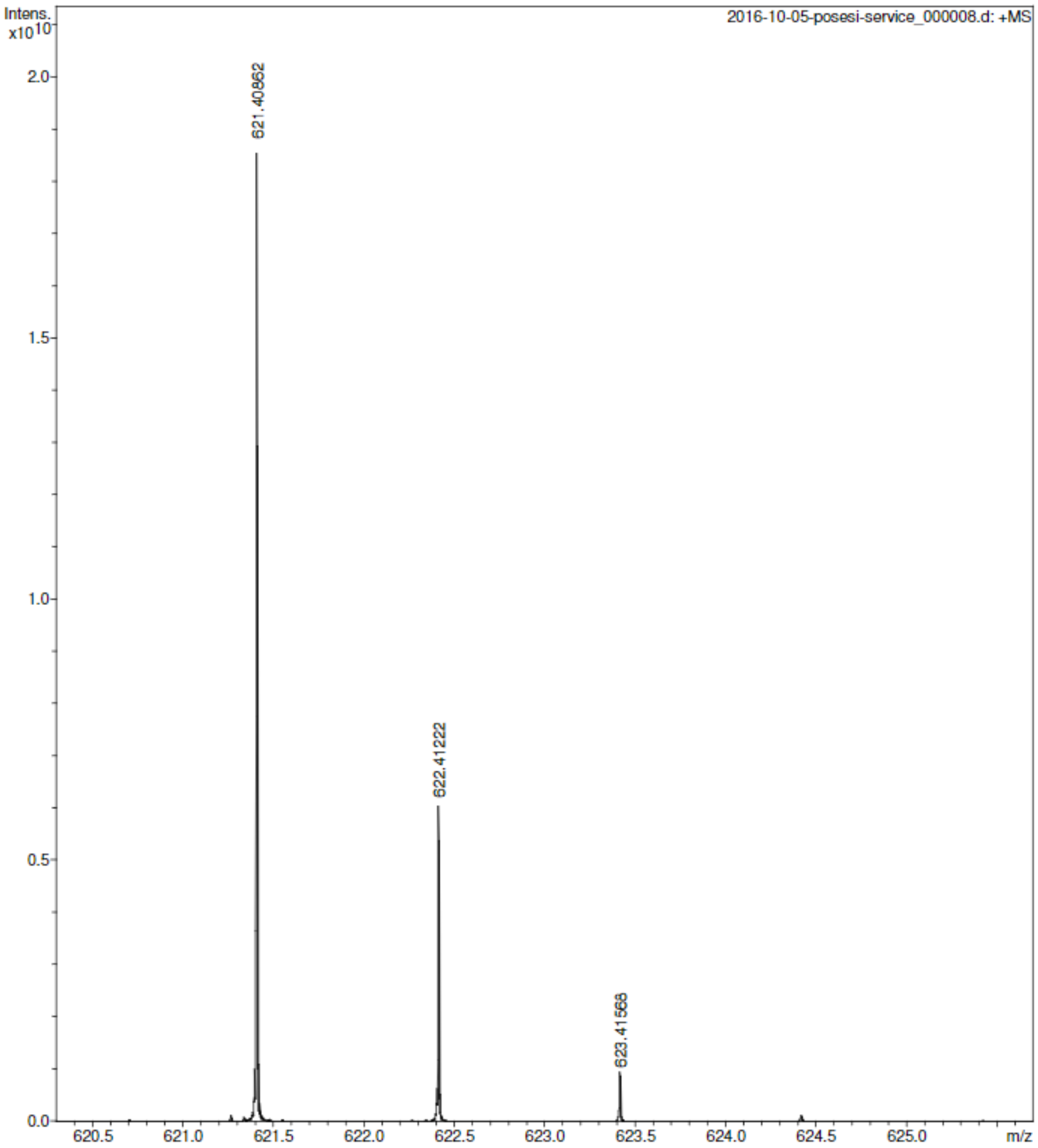

Figure S1. High resolution ESI mass spectrum of F12. F12 (~100 µg) was dissolved in 100% acetonitrile for injection and analysis via high resolution ESI mass spectrometry (m/z 621.4086). Results identified chemical formula $C_{32}H_{58}N_2NaO_8$ $[M+Na]^+$ for the adduct ion as the only possible formula assuming C, H, N, O, Na (0-1), even electron configuration and mass error <2ppm. This adduct ion corresponds to an average mass of 598.822 g/mol for the natural product.

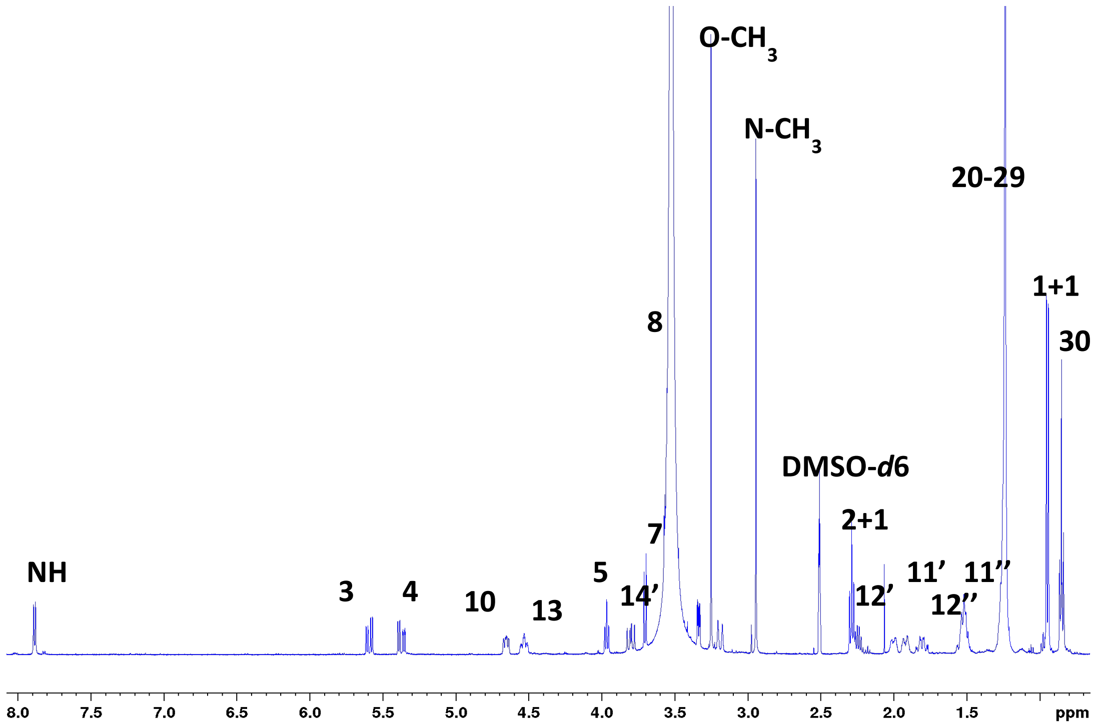

Figure S2. ^{1}H NMR spectrum for F12. F12 (~1mg) was dissolved in 45uL of DMSO-*d*6. ^{1}H NMR spectrum was recorded on a Bruker AVANCE III 500 NMR spectrometer.

**Structure**	**F12**<br>**DMSO-*d6***	**Bengamide B[a]**<br>**$CDCl_3$**
**Carbon Number**	**δ_C**<br>**(ppm)**	**δ_C**<br>**(ppm)**
17	172.65	173.02
16	172.31	172.15
9	170.33	171.78
3	138.56	141.48
4	128.04	125.40
8	81.95	80.83
5	73.22	74.32
6	72.94	72.86
7	71.18	72.33
13	69.62	69.17
O-CH_3	57.77	60.06
14	52.7	53.36
10	51.16	51.33
N-CH_3	36.11	36.40
18	34.04	34.35
12	32.5	32.68
28	31.74	31.92
2	30.59	30.80
20	29.49	29.68
21	29.455	29.65
22	29.45	29.60
23	29.4	29.45
24	29.29	29.35
25	29.15	29.24
26	29.1	29.10
27	28.82	29.00
11	28.61	
19	24.81	24.78
29	22.75	22.69
1	22.64	22.22
15	22.54	22.11
30	14.38	14.12

[a]Data taken from[44]

Table S2. ^{13}C NMR spectrum of F12.

^{1}H NMR (500MHz, DMSO-*d6*): δ 7.89 (d, *J* = 6.6; 1H), 5.59 (ddd, J = 15.6, 6.6, 1.0, 1H), 5.37 (ddd, J = 15.6, 6.7, 1.3, 1H), 4.66 (dd, J = 10.8, 6.6, 2H), 4.54 (dddd, J = 10.1, 1.9, 2.0, 1H), 3.97 (dd, J = 7.2, 6.7, 1H), 3.71 (d, 7.1, 2H), 3.80 (dd, J = 14.8, 10.1, 1H), 3.57 (dd, J = 7.2, 2.7, 1H), 3.34 (dd, J = 6.6, 2.6, 1H), 3.25 (s, 3H), 3.20 (dd, J = 14.8, 2.0, 2.0, 1H), 2.94 (s, 3H), 2.29 (t, J = 7.3, 2H), 2.15 (qqd, J = 6.7, 6.7, 6.6, 1H), 2.0 (m, 1H), 1.92 (m, 1H), 1.81 (m, 1H), 1.56-1.48 (overlapping m, 3H), 1.30-1.19 (overlapping m, 20H), 0.94(7) (d, J = 6.7, 3H), 0.94(5) (d, J = 6.7, 3H), 0.85 (app. t, 6.7, 3H). ^{13}C NMR (125MHz, DMSO-*d6*): δ 172.65. 172.31, 170.33, 138.56, 128.04, 81.95, 73.22, 72.94, 71.18, 69.62, 57.77, 52.7, 51.16, 36.11, 34.04, 32.5, 31.74, 30.59, 29.49, 29.455, 29.45, 29.4, 29.29, 29.15, 29.1, 28.82, 28.61, 24.81, 22.75, 22.64, 22.54, 14.38.

Figure S3. ^{1}H and ^{13}C NMR signals. F12 (~1mg) was dissolved in 45uL of DMSO-*d6*. ^{1}H and ^{13}C NMR spectra were recorded on a Bruker AVANCE III 500 NMR spectrometer.

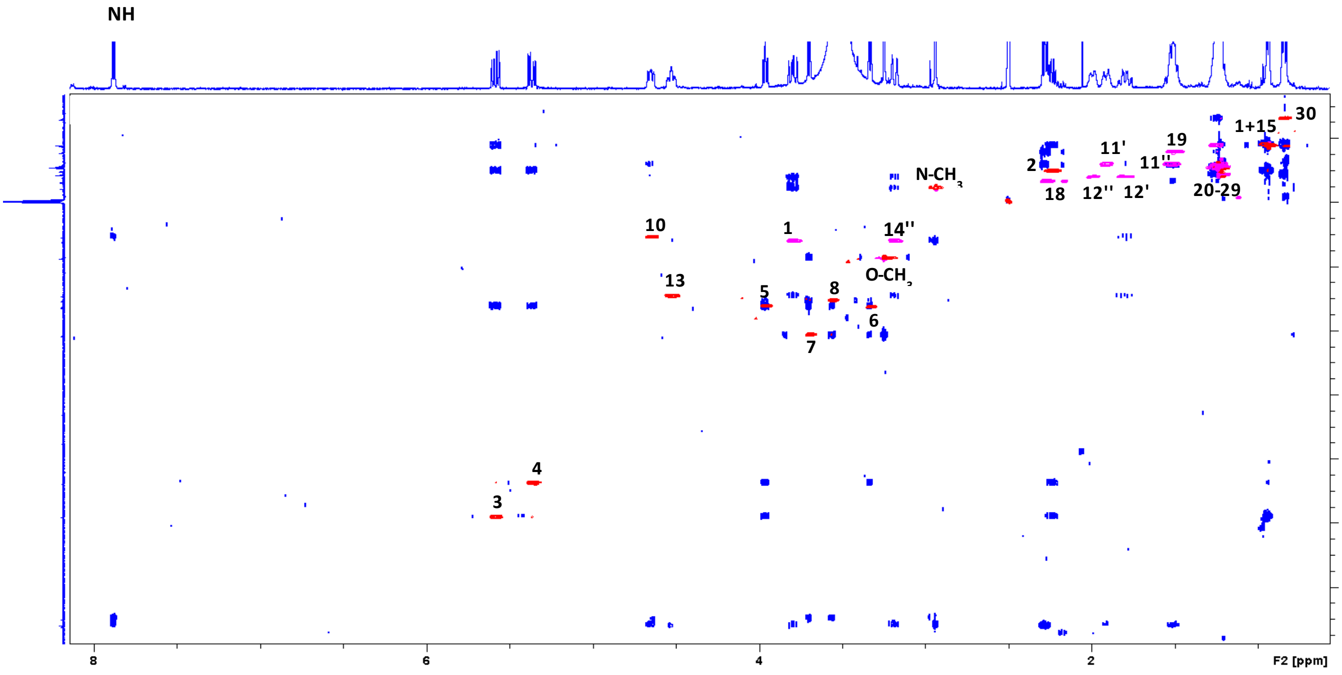

Figure S4. Stacked 2D NMR 1H-13C HSQC and HMBC spectra of F12. ^{1}H NMR (500 MHz, DMSO-d_6) and ^{13}C NMR (125 MHz, DMSO-d_6) of F12 (~1mg) were analysed using stacked heteronuclear single quantum correlation (HSQC) and heteronuclear multiple bond correlation (HMBC). Red cross-peaks indicate CH or CH_3 HSQC correlations. Pink cross-peaks indicate CH_2 HSQC correlations. Blue cross-peaks indicate HMBC correlations.
